# Individual and Contextual Determinants of Regional Variation in Prescription Drug Use: An Analysis of Administrative Data from British Columbia

**DOI:** 10.1371/journal.pone.0015883

**Published:** 2010-12-29

**Authors:** Steven G. Morgan, Colleen M. Cunningham, Gillian E. Hanley

**Affiliations:** 1 Centre for Health Services and Policy Research, University of British Columbia, Vancouver, British Columbia, Canada; 2 School of Population and Public Health, University of British Columbia, Vancouver, British Columbia, Canada; Yale University School of Medicine, United States of America

## Abstract

**Background:**

Increasing attention is being paid to variations in the use of prescription drugs because their role in health care has grown to the point where their use can be considered a proxy for health system performance. Studies have shown that prescription drug use varies across regions in the US, UK, and Canada by more than would be predicted based on age and health status alone. In this paper, we explore the determinants of variations in the use of prescription drugs, drawing on health services theories of access to care.

**Methods:**

We conducted a cross-sectional analysis using population-based administrative health care data for British Columbia (BC), Canada. We used logistic and hierarchical regressions to analyze the effects of individual- and area-level determinants of use of prescriptions overall and rates of purchase of prescriptions from five therapeutic categories representing a range of indications: antihypertensives, statins, acid reducing drugs, opioid drugs, and antidepressants. To indicate the relative scale of regional variations and the importance of individual- and area-level variables in explaining them, we computed standardized rates of utilization for 49 local health areas in BC.

**Results:**

We found that characteristics of individuals and the areas in which they live affect likelihood of prescription drug purchase. Individual-level factors influenced prescription drug purchases in ways generally consistent with behavioral models of health services use. Contextual variables exerted influences that differed by type of drug studied. Population health, education levels, and ethnic composition of local areas were associated with significant differences in the likelihood of purchasing medications. Relatively modest regional variations remained after both individual-level and area-level determinants were taken into account.

**Conclusions:**

The results of this study suggest that individual- and area-level factors should be considered when studying variations in the use of prescription drugs. Some sources of such variations, including individual- and area-level socioeconomic status, warrant further investigation and possible intervention to address inequities.

## Introduction

Many studies have shown that medical and surgical practices vary across regions and/or populations by more than would be expected based on medical needs alone [1], [2], [3], [4], [5], [6], [7], [8]. These variations are increasingly viewed as indicators of health system performance and of the quality of care received by populations because they can be costly in terms of both financial expense and health outcomes [6], [7], [8]. Although much more is known about regional variations in the use of other health care services, increasing attention is being paid to variations in the use of prescription drugs because their role in health care has grown to the point where their use can be considered a proxy for health system performance. Studies have shown that prescription drug use varies across regions in the US, UK, and Canada by more than would be predicted based on age and health status alone [9], [10], [11], [12], [13]. Some of these studies indicate that area-level factors such as ethnic composition, physician supply, and socioeconomic status may partially explain regional variations in medicine use [10], [11], [13].

We have previously demonstrated that prescription drug use and cost varies across Canadian provinces[14], [15] and across regions within provinces[16], [17] by more than might be expected based on variations in population age and health status. Using administrative health care data that cover virtually all of the 4 million residents of British Columbia (BC), Canada, and informed by health services research theories concerning access to care, we aimed to test hypotheses about the extent to which variations in the use of medicines may be explained by the characteristics of individuals and/or by the characteristics of the communities in which they live. We first tested models of prescription drug use that included only individual-level factors such as age, sex, health status, and income. We then added area-level contextual factors such as primary care supply, population health, and ethnic concentration. After testing these in logistic regression analyses, we illustrated the effects that accounting for individual- and area-level characteristics had on measures of regional variations in prescription drug use using summary measures of variation.

We conducted our analysis using population-based administrative health care datasets covering virtually all of the 4 million residents of BC. Because the extent of variation in the use of prescription drugs (across regions and population subgroups) is greater for specific therapeutic categories than for all categories combined [14], [15], [16], [17], we studied variations in the prevalence of prescription drug purchases from five therapeutic categories representing a range of indications.

### Context

BC is a geographically, economically, and culturally diverse province of Canada, with a population of approximately 4.1 million (data for 2006). The province covers an area nearly the size of France and Germany combined; however, approximately 85% of the population is concentrated in just a few urban areas, especially in southern regions close to the Canada-US border. Over half of the residents of BC (∼2.1 million) live in the metropolitan area of Vancouver alone. As of the 2006 Census, the median income per family in BC was CAD$62,000, with the major economic activities being financial services (25% of provincial GDP), manufacturing (8%), transportation (6%), and energy (5%) [18]. In 2006, an estimated 27% of the population of BC (∼1.1 million people) were immigrants to Canada, more than half of whom emigrated from Asia [19].

Residents of BC are covered under a universal public health insurance system that provides full first-dollar coverage for medical and hospital care. BC residents may also register for the Fair PharmaCare program, which provides public subsidy of prescription drug costs that exceed deductibles set at certain percentages of household income. Deductibles for persons born prior to 1939 are relatively low; for all other residents, deductibles are set at gradually increasing percentages of income and reach a maximum of 3% of household income for all households with incomes exceeding CAD$30,000. Because of the structure of the program, a majority of households in BC face considerable deductibles before any public drug benefits are provided. Many residents have supplementary (usually employment-related) private health insurance that covers prescription drugs. Private insurance plans are estimated to cover approximately 40% of total prescription drug expenditures, public programs another 40%, and out-of-pocket payments are estimated to account for 20% [20].

## Methods

### Ethics statement

With permission from data stewards at the BC Ministry of Health Services and the College of Pharmacists of BC, and the approval of the University of British Columbia research ethics board, we conducted this study using de-identified linked datasets from Population Data BC and BC PharmaNet. Informed consent from patients is not required for use of these de-identified administrative databases.

### Framework

We based our analysis on a modified version of the Andersen, Newman, Aday framework with three dimensions of patient characteristics that act as predictors of health care use: predisposing characteristics, such as age and sex; enabling resources, such as income and insurance; and needs, such as diagnosed illness [21], [22], [23]. Based on a model described by Phillips and colleagues, we included contextual variables that describe the setting in which use of care occurs [24]. In particular, we explored community-level factors such as the availability of primary care providers, overall population health, average incomes, rates of post-secondary education, and ethno-cultural mix.

### Data and cohort

Our datasets contained records of every filled prescription, hospital discharge, and fee-for-service medical visit during calendar year 2006 for every resident of BC except status Indians, veterans, and Royal Canadian Mounted Police, who are federally covered for healthcare (∼5% of the total provincial population). The BC PharmaNet database tracks every prescription dispensed from community pharmacies and long-term care facilities, regardless of patient age or insurance status; however, it excludes prescription drugs used within acute care hospitals.

To ensure complete data capture for study subjects, we excluded any resident who lived in BC for fewer than 275 days in 2006 (∼6% of the population). To provide for reasonable comparability of health system and social contexts, we excluded rural and remote areas of BC (∼6.5% of the population). The resulting cohort contained 3.92 million residents (∼84.1% of the total provincial population, 89.6% of the population within the non-rural regions studied).

### Outcomes Variables

In previous work, we found that the most significant factor contributing to regional variations in the cost and volume of prescription medicines used is variation in the likelihood that individuals will fill one or more prescriptions [16], [17]. Our primary outcomes in this study were therefore period prevalence measures of prescription drug purchases during the calendar year of 2006. We created binary outcomes variables indicating whether a person filled one or more prescriptions from each of five therapeutic categories: antihypertensives, statins, acid-reducing drugs, opioid drugs, and antidepressants. These categories represent a range of primary indications and include some that may be deemed less discretionary than others (e.g., antihypertensives versus antidepressants); non-medical factors at the individual and area level may have a greater impact on use of discretionary medicines. Appendix S1 contains a list of the specific types of drug included in each drug class analyzed.

### Individual-Level Explanatory Variables

For every person in our sample, we constructed measures of general and condition-specific health care needs using the Johns Hopkins University ACG Case-Mix System [25]. The ICD-9/ICD-10 diagnostic codes for this came from hospital discharge records (up to 25 codes per discharge) and billings for fee-for-service medical visits (one code per visit). We used a count of the Aggregated Diagnostic Groups (ADGs) as a general health status measure: a higher count of ADGs indicates greater clinical complexity. We used Expanded Diagnostic Clusters (EDCs) to indicate the presence of common indications (e.g., hypertension) for each drug class studied (e.g., antihypertensives); Appendix S1 contains a list of the EDCs used for each drug class.

Our individual-level predisposing variables were age and sex, while our individual-level enabling variable was relative income ranking (in deciles). For approximately 80% of our sample, income rankings were based on household-specific tax-return data maintained by the province for the purpose of administering the Fair PharmaCare program. For the remaining population, income ranks were assigned based on neighborhood income estimates provided by Statistics Canada [26].

### Area-level Explanatory Variables

Our geographic units of analysis are local health areas (LHAs), used for planning health services delivery in BC. The 49 predominantly urban LHAs studied ranged in population size from 8,683 to 298,253 (median  = 45,744). To gauge population health within each LHA, we obtained from BC Stats the average potential years of life lost (PYLL) due to natural causes between 2003 and 2007. We used the share of 2006 Census respondents that reported being Chinese, South Asian, or another visible minority to provide information about the ethnic composition of LHAs. (Area-level immigration was highly correlated with prevalence of Chinese ethnicity and therefore not included in our models.) To measure LHA socioeconomic characteristics, we used 2006 Census data on average household incomes and percentage of population over age 20 with some post-secondary education. Finally, we obtained published estimates of the number of full-time equivalent primary care physicians per 100,000 residents within each LHA during 2006 to measure primary health care supply [27].

### Statistical models

For each outcome, we ran two regression models: the first controlled for individual-level independent variables only and the second controlled for individual- and area-level independent variables. We used generalized estimating equation (GEE) models to account for clustering at the LHA. Owing to the large size of our database, we were unable to run those models on the full dataset (3.293 million observations). We therefore ran the multi-level GEE models on a 2% random sample of the full dataset (62,459 observations) and logistic regressions on the sample and full datasets. Estimates of standard errors in the logistic models were adjusted for clustering of individuals within LHAs. We compared the goodness of fit for models with and without the area-level variables using log-likelihood ratio tests.

To determine the effects that accounting for individual- and area-level characteristics have on measures of regional variation in prescription drug use, we used the regression models to compute standardized rates of utilization for each LHA. The standardized utilization rate can be interpreted as what the provincial rate would be if people in all areas of the province used medicines (or other health services) at the same age-, sex-, health-, and income-adjusted rates as residents in the specified LHA and, when area-level variables are included, if all areas of the province had the same contextual characteristics as the LHA in question. Using these standardized rates, we computed summary statistics and coefficients of variation to gauge the impact of the area-level contextual variables on the measured variation across LHAs. All analyses were run on STATA version 10.0 (StataCorp LP).

## Results

Table 1 describes the individual- and area-level characteristics of our study population. Our study sample included 3,292,605 individuals living in 49 non-rural LHAs. The sample age (40.2) and sex (51% female) distribution was equal to census figures for the province. Residents in our sample had diagnoses in their administrative health records that indicated an average of 3.2 ADGs in 2006. The LHAs included in this study varied moderately (coefficient of variation, CV>0.20) in terms of population health (PYLL), average income, and primary health care supply; LHAs varied considerably (CV>1.00) in terms of percentages of Chinese and South Asian populations. Of the drug types studied, antihypertensives (15%), opioids (12%), and antidepressants (10%) were most commonly used.

**Table 1:** Characteristics of the study population.

Variable	Result	CV
Sample size	3,292,605	
**Individual-level characteristics**		
Female share	0.51	
Age mean	40.2	0.55
Overall needs, mean # of ADGs	3.2	0.88
**Contextual (area-level) characteristics**		
Potential years life lost, mean	32.8	0.25
Chinese share	0.11	1.27
South Asian share	0.07	1.29
Other minority share	0.09	0.67
Post-secondary share	0.62	0.13
Average income, $1000s	69.1	0.20
Primary care supply, mean FTE/100,000 residents	8.6	0.27
**Use of prescription drugs (% province-wide)**		
Antihypertensives	0.15	2.40
Statins	0.07	3.71
Acid reducing drugs	0.08	3.38
Opioids	0.12	2.67
Antidepressants	0.1	3.00

CV  =  coefficient of variation.

### Determinants of utilization

Likely because the correlation of errors within clusters was low, results of the GEE models on a 2% sample of the data and logistic models were virtually identical – all adjusted odds ratios were equal to the third or fourth decimal and no tests of statistical significance changed [28]. Table 2 lists results from the full sample logistic regressions for prescription drug purchases by therapeutic category. All five of the models containing only individual-level variables suggest that individual-level health needs, predisposing factors and enabling factors are significant for explaining variations in prescription drug use (p<0.001). Moreover, individual-level health needs and predisposing factors had expected impacts on the likelihood of category-specific prescription purchases. There was a u-shaped relationship between income and the likelihood of purchasing drugs from each class studied except for opioids (for which there was a negative income gradient).

**Table 2:** Adjusted odds ratios for the likelihood of purchasing one or more prescription from specific therapeutic categories in 2006, non-rural local health areas of British Columbia.

	Antihypertensives		Statins		Acid reducing drugs		Opioid drugs		Antidepressants	
	A	B	A	B	A	B	A	B	A	B
**Needs**										
Overall needs (# of ADGs)	1.069**	1.070**	1.095**	1.094**	1.262**	1.263**	1.242**	1.245**	1.143**	1.146**
Treatment-specific need^1^	27.658**	27.817**	7.438**	7.373**	18.165**	17.639**	3.223**	3.129**	7.840**	7.751**
**Predisposing (sex, age** 2 **)**										
Female (Male = ref)	1.193**	1.196**	0.663**	0.665**	1.094**	1.098**	0.818**	0.819**	1.432**	1.438**
Age 10–14 (50–54 = ref)	0.023**	0.023**	0.001**	0.001**	0.110**	0.109**	0.134**	0.132**	0.097**	0.095**
Age 30–34 (50–54 = ref)	0.223**	0.226**	0.073**	0.073**	0.396**	0.400**	0.905**	0.920**	0.587**	0.598**
Age 70–74 (50–54 = ref)	2.988**	2.970**	4.411**	4.475**	1.788**	1.785**	0.762**	0.756**	0.795**	0.785**
Age 90–94 (50–54 = ref)	4.069**	4.072**	1.123*	1.165**	1.645**	1.661**	0.566**	0.561**	0.887*	0.869**
**Enabling (income** 2 **)**										
Lowest decile (middle = ref)	1.103**	1.135**	1.188**	1.188**	1.337**	1.356**	1.276**	1.317**	1.569**	1.627**
3rd income decile (middle = ref)	0.952*	0.969	1.042*	1.021	1.023	1.031*	1.003	1.043	0.958	1.005
7th income decile (middle = ref)	1.039*	1.037**	1.067**	1.077**	1.018	1.023	0.992	0.980*	1.001	0.989
Highest income decile (middle = ref)	1.157**	1.187**	1.215**	1.264**	1.076**	1.120**	0.947*	0.966*	1.001	1.023
**Contextual (area-level)**										
Potential years life lost		1.002		0.996*		1.001		1.004**		1.006**
Chinese share		0.999		1.001		0.999		0.988**		0.987**
South Asian share		0.997		1.008**		1.000		0.997		0.996*
Other minority share		0.992*		0.999		0.993**		0.995*		0.990**
Post-secondary share		0.990**		0.985**		0.997		0.994*		0.998
Average income ($1000s)		1.001		1.001		0.997**		1.000		0.999
Primary care supply		1.012		1.008		0.992		0.999		1.006
C-statistic (%)	95.65	95.67	91.05	91.12	86.24	86.29	80.47	80.79	87.37	87.66

A  =  individual level, B  =  individual and area level

1  =  table shows odds ratio for only one Expanded Diagnostic Clusters (EDC) from in each category-specific logistic regression model. Appendix S1 contains a complete list of diagnoses used for each category-specific analysis.

2  =  table shows only examples of the 20 age groups and 10 income groups.

* =  significant at or below p = 0.05.

** =  significant at or below p = 0.01.

Adjusted odds ratios on individual-level variables did not change significantly when area-level variables were added to the model, and the models containing both individual- and area-level variables were better fit (LR test, p<0.001), though the change in the predictive power of the model was very modest (small changes in C-statistics). Area-level variables had different impacts on the likelihood of purchasing drugs of different types. The level of population health needs (PYLL) was positively associated with the likelihood of purchasing antidepressants and opioid drugs and negatively associated with the likelihood of purchasing statins. Higher concentrations of ethnic minorities in LHAs were generally associated with a lower likelihood of prescription purchases, but the results varied by drug category. For example, the share of the local population that identified as Chinese was negatively associated with the likelihood of purchasing antidepressants and opioid drugs but not significantly associated with the likelihood of purchasing antihypertensives, statins, or acid-reducing drugs. Area-level supply of primary care physicians was not significantly associated with the likelihood of purchasing any of the five types of medicine studied.

### Impact on measures of regional variations

Table 3 lists summary statistics describing the distribution across LHAs of prevalence rates for prescription drug purchases from the five therapeutic categories. The table summarizes variations in crude rates of medicine use, as well as standardized rates based on predictions from the logistic regression with adjustments for individual-level determinants and from the logistic regression with adjustments for individual- and area-level determinants. The magnitude of variation in crude rates of prescription purchases across regions was comparable for all five drug classes studied. The extent to which measured regional variation was attenuated by the addition of individual- and area-level predictors of drug use differed by specific type of prescription drug.

**Table 3:** Summary statistics for regional variations in rates of purchasing one or more prescription from specific therapeutic categories in 2006, non-rural local health areas of British Columbia.

	Min	Median	Max	Max-Min Ratio	Inter-quartile ratio	CV
**Antihypertensives**						
Crude	0.08	0.16	0.23	2.67	1.27	0.18
Adjusted, individual	0.14	0.15	0.18	1.29	1.11	0.07
Adjusted, individual and area	0.13	0.15	0.16	1.22	1.05	0.04
**Statins**						
Crude	0.04	0.07	0.11	2.91	1.20	0.20
Adjusted, individual	0.06	0.07	0.09	1.53	1.20	0.11
Adjusted, individual and area	0.06	0.07	0.08	1.36	1.11	0.07
**Acid reducing drugs**						
Crude	0.05	0.08	0.11	2.20	1.16	0.14
Adjusted, individual	0.06	0.08	0.11	1.72	1.14	0.11
Adjusted, individual and area	0.06	0.08	0.10	1.57	1.08	0.08
**Opioid drugs**						
Crude	0.07	0.13	0.16	2.28	1.22	0.18
Adjusted, individual	0.08	0.12	0.16	2.06	1.21	0.16
Adjusted, individual and area	0.10	0.11	0.13	1.31	1.10	0.07
**Antidepressants**						
Crude	0.05	0.11	0.14	2.60	1.26	0.19
Adjusted, individual	0.06	0.11	0.14	2.23	1.22	0.17
Adjusted, individual and area	0.08	0.09	0.11	1.39	1.07	0.07

The addition of individual-level variables to create adjusted measures of prevalence reduced measures of regional variation in the purchase of each type of prescription drug; however, measured variation fell most notably for antihypertensives and statins when individual-level factors were accounted for. The CVs for these categories changed from 0.18 to 0.07 and 0.20 to 0.11, respectively. The addition of individual-level variables had the least effect on measures of regional variation in the purchase of antidepressants and opioid drugs. In contrast, while the addition of area-level variables to the adjustment model reduced measured variations for all drug types, the effects of area-level variables were greatest for measured variation in the use of antidepressants and opioid drugs - the CVs for these categories changed from 0.17 to 0.07 and 0.16 to 0.07, respectively, with the addition of area-level variables.

## Discussion

Our results provide evidence indicating that the characteristics of individuals and of the areas in which they live affect likelihood of prescription drug use and that these characteristics may help to explain regional variations in prescription drug use. Beyond the expected contributions of individual-level factors, which influenced prescription drug use in ways generally consistent with behavioral models of health services use, area-level variables were important determinants of regional variations in use. For example, area-level measures of population health and socioeconomic status affected the likelihood of purchasing several types of prescription drugs; similarly, area-level concentrations of one or more ethnic minorities were negatively associated with purchases of all types of medicine studied except statins. Because these area-level factors vary considerably across regions, their addition to statistical models significantly reduced measures of regional variations in (adjusted) rates of prescription drug use.

Our measures of variation that standardize for individual-level factors only are comparable to health status stratified results that Dubois and colleagues documented for 1998/99 in California [29]. Though no study has factored area-level characteristics into measures of regional variations, previous studies have found similar influences concerning area-level factors and use of specific prescription drugs. In a study of stimulant use among insured children in the US, Cox and colleagues found average income and the proportion of whites within neighborhoods were positively associated with the likelihood of stimulant use but that there was no association between physician supply and stimulant use [13]. Analyzing rates of treatment for anxiety and depression in 39 deprived areas of England at an ecological level, Goyder and colleagues found that prescribing rates were positively associated with the supply of general practitioners and negatively associated with the share of the population for whom English was not their first language [11]. Finally, also using a form of ecological analysis, Ward and colleagues found a negative association between statin prescribing and the share of ethnic minorities in the populations served across 132 GP practices in northwest England [10].

### Limitations

Several limitations of our study should be noted. For certain constructs, we were unable to generate corresponding measures at the individual and area level. Most notably, we were unable to identify ethnicity and immigration at the individual level for this study population because such data are not routinely collected in Canada. Coefficients on our area-level ethnicity variables may therefore have explanatory power in our models because they serve, in part, as proxies of individual-level ethnicity. Additionally, because our health status measures were constructed based on data derived from contacts with the health care system, our research methods may understate the level of health needs for groups who experience economic, cultural, or other demonstrable barriers to accessing health care. In analyses done to test for this, we did not find significant evidence of access barriers to primary care (results not shown).

### Conclusions

The results of this study suggest that individual- and area-level factors should be taken into consideration when studying variations in the use of prescription drugs. This is not to suggest that such determinants of variation should be used to mask what might otherwise be important disparities in use of treatments across population subgroups. Instead, the analysis of variation in medicine use should be used to illuminate determinants so that systemic disparities in access to health services may be addressed or planned for as appropriate [23]. At the individual level, for example, the u-shaped income gradients found in this study suggest that the income-based system of drug coverage in BC may create prescription drug access barriers (particularly for lower-middle-income households) that deserve further investigation. At the contextual level, our findings concerning education, health status, and concentrations of ethnic minorities all point to areas requiring more in-depth investigation and possible intervention to address inequities.

## Supporting Information

Appendix S1 **Details regarding the drug categories and category-specific indications of need used in statistical models.** Lists the names of the relevant therapeutic categories, the types of drugs in each therapeutic category and the Expanded Diagnostic Clusters (EDCs) used to adjust for specific indications within each category.
(DOC)
