# Supplementary material for: Individual and Contextual Determinants of Regional Variation in Prescription Drug Use: An Analysis of Administrative Data from British Columbia
Source: PLoS One. 2010 Dec 29;5(12):e15883. doi: 10.1371/journal.pone.0015883 (PMC3012101; doi:10.1371/journal.pone.0015883)
Supplement: Appendix S1 — Details regarding the drug categories and category-specific indications of need used in statistical models. Lists the names of the relevant therapeutic categories, the types of drugs in each therapeutic category and the Expanded Diagnostic Clusters (EDCs) used to adjust for specific indications within each category. (DOC) [file pone.0015883.s001.doc]

#### Appendix S1 : Details regarding the drug categories and category-specific indications of need used in statistical models

| **Name of therapeutic category** | **Types of drug in therapeutic category** | **Expanded Diagnostic Clusters (EDCs) used to adjust for specific indications** |
| --- | --- | --- |
| **Acid reducing drugs** | Histamine (2) Receptor Antagonists (e.g. ranitidine); Proton pump inhibitors (e.g. omeprazole) | GAS08: Gastroesophageal reflux; GAS06: peptic ulcer disease |
| **Antidepressants** | Nonselective monoamine reuptake inhibitors (e.g. amitriptyline); Monoamine oxidase inhibitors (e.g. phenelzine); Trazodone ; Selective serotonin reuptake inhibitors (e.g. citalopram); Venlafaxine ; Mirtazapine; Moclobamide; Bupropion | PSY01: Anxiety, neuroses; PSY09: Depression; PSY10: Psychological signs and symptoms |
| **Antihypertensives** | Thiazide diuretics (e.g. hydrochlorothiazide); Beta blocking agents (e.g. atenolol); Calcium channel blockers (e.g. nifedipine); Angiotensin converting enzyme inhibitors (e.g. ramipril); Angiotensin receptor blockers (e.g. losartan) | CAR14: Hypertension, w/o major complications; CAR15: Hypertension, with major complications; CAR01: cardiovascular signs and symptoms; CAR03: Ischemic heart disease; CAR05: Congestive heart failure; CAR07: Cardiomyopathy; CAR10: Generalized atherosclerosis; CAR12: Acute myocardial infarction; END06: Type 2 diabetes, w/o complication; END07: Type 2 diabetes, w/ complication; END08: Type 1 diabetes, w/o complication; END09: Type 1 diabetes, w/ complication |
| **Opioid analgesics** | Opioids (e.g. morphine) | NUR02: Headaches; RHU01: Autoimmune and connective tissue diseases; RHU02: Gout; RHU03: Arthropathy; MAL01-MAL18 - all malignancies; MUS01 Musculoskeletal signs and symptoms; MUS02: Acute sprains and strains; MUS03: Degenerative disc disease; MUS04 Fractures; MUS 08: Fractures and dislocations; MUS09: Joint disorders, trauma related; MUS10: Fracture of neck of femur; MUS13: cervical pain syndrome; MUS14: low back pain; MUS15: Bursitis, synovitis, tenosynovitis; MUS17: musculoskeletal disorders, other |
| **Statins** | Statins (e.g. atorvastatin) | CAR11: Disorders of lipoid metabolism; CAR12: Acute myocardial infarction; CAR03: Ischemic heart disease; NUR05: Cerebrovascular disease ; CAR10: Generalized atherosclerosis |
